# Polygenic Risk Score Improves Melanoma Risk Assessment in a Patient Cohort from the Veneto Region of Italy

**DOI:** 10.3390/biology13110954

**Published:** 2024-11-20

**Authors:** Stefania Pellegrini, Thomas P. Potjer, Paola Del Bianco, Antonella Vecchiato, Alessio Fabozzi, Luisa Piccin, Debora Tonello, Nienke van der Stoep, Emily Tinsley, Maria Teresa Landi, Mark M. Iles, Chiara Menin

**Affiliations:** 1 Immunology and Molecular Oncology Unit, Veneto Institute of Oncology IOV-IRCCS, 35128 Padua, Italy; debora.tonello@iov.veneto.it (D.T.); chiara.menin@iov.veneto.it (C.M.); 2 Department of Clinical Genetics, Leiden University Medical Center, 2333 ZC Leiden, The Netherlands; t.p.potjer@lumc.nl (T.P.P.); n.van_der_stoep@lumc.nl (N.v.d.S.); 3 Clinical Trials and Biostatistics Unit, Veneto Institute of Oncology IOV-IRCCS, 35128 Padua, Italy; paola.delbianco@iov.veneto.it; 4 Soft-Tissue, Peritoneum and Melanoma Surgical Oncology Unit, Veneto Institute of Oncology IOV-IRCCS, 35128 Padua, Italy; antonella.vecchiato@iov.veneto.it; 5 Oncology 3 Unit, Department of Oncology, Veneto Institute of Oncology IOV-IRCCS, 35128 Padua, Italy; alessio.fabozzi@iov.veneto.it; 6 Oncology 2 Unit, Department of Oncology, Veneto Institute of Oncology IOV-IRCCS, 35128 Padua, Italy; luisa.piccin@iov.veneto.it; 7 Leeds Institute for Data Analytics, University of Leeds, Leeds LS2 9NL, UK; emilytinsley774@gmail.com (E.T.); m.m.iles@leeds.ac.uk (M.M.I.); 8 Division of Cancer Epidemiology and Genetics, National Cancer Institute, Bethesda, MD 20892-8322, USA; landim@mail.nih.gov; 9 NIHR Leeds Biomedical Research Centre, Leeds Teaching Hospitals NHS Trust, Leeds LS7 4SA, UK

**Keywords:** melanoma, genetic predisposition, polygenic risk score, risk factors, multiple primary melanoma

## Abstract

Recent genome-wide association studies (GWASs) have identified many single nucleotide polymorphisms (SNPs) that alone weakly affect melanoma risk, but their combined effect on a polygenic risk score (PRS) can have a far bigger impact on estimating risk. However, the PRS is not yet at the stage of being utilized in clinical practice, and further evidence is needed. In this study, 270 melanoma patients fulfilling the criteria for a suspected genetic predisposition but with a negative genetic test for high/medium-penetrance genes were genotyped for 57 SNPs selected in previous GWASs to construct a PRS model. We found a significantly higher mean PRS_57_ in all melanoma cases than in controls (0.58 vs. 0.00, *p* < 0.001), and the mean PRS_57_ in multiple primary melanoma cases was twice that in single melanoma cases (0.689 vs. 0.362, *p* = 0.025). Interestingly, our results confirm the association of the PRS_57_ not only with other melanoma risk factors but also with a younger age at diagnosis. This evidence supports the potentially powerful discriminative role of PRS in the selection of high-risk patients who should undergo stricter surveillance protocols.

## Simple Summary

Identifying individuals at high risk of developing melanoma is a crucial starting point for prevention and early detection, which are still the best treatment options for cutaneous melanoma. However, despite the scientific advances, unraveling the missing heritability of melanoma-prone patients without an inherited pathogenic variant in a known melanoma predisposition gene remains a great challenge. For this reason, we aim to define a polygenic risk score (PRS) that could be associated with the risk of developing melanoma, also considering non-genetic risk factors. Interestingly, we found that individuals with a high PRS are more likely to develop not only one melanoma but also subsequent melanomas. Moreover, our findings are indicative of an association of the PRS with some individual phenotypic/behavioral characteristics and also with a younger age at diagnosis. The PRS, as a new tool to be included in clinical practice, could help stratify patients according to their individual risk of developing melanoma, improving early diagnosis, known to be a key factor in determining the prognosis of patients.

## 1. Introduction

Melanoma is a cancer arising from the uncontrolled proliferation of melanocytes. Even though these neural crest-derived cells are primarily involved in skin pigmentation and skin protection from UV-induced DNA damage, they can develop into malignant melanoma, either spontaneously or in response to external triggers (e.g., sun exposure) [1]. In normal melanocytes, melanin pigment is usually synthesized in a highly controlled process, thus playing a protective role against environmental threats and UVR-induced transformations. However, under certain conditions, melanin can also exhibit phototoxic activity due to its atypical physico- and photo-chemical properties [2]. Therefore, uncontrolled melanogenesis could critically contribute to the malignant transformation of melanocytes. In 1998, Whiteman proposed the compelling “two divergent pathways” hypothesis, which sought to explain the combined impact of host and environmental factors on melanoma development [3]. Furthermore, in 2018, the World Health Organization (WHO) introduced a new melanoma classification based on the progression model, in which nine main pathways attributable to two groups, UV-related or not, underlie the different histological types of melanocytic tumors with intermediate lesions as simulators and/or precursors of melanoma [4].

Melanoma incidence is increasing worldwide, especially in countries with a predominantly fair-skinned population and a high ultraviolet (UV) index [5,6,7]; this supports the implication of host characteristics (i.e., number of common and atypical nevi, phenotypic traits, a positive personal or familial history of melanoma and/or other tumor types) and environmental factors (UV exposure pattern) on the etiology of melanoma and thus on individual melanoma risk [8,9,10,11]. Even though some of these risk factors are well defined, there are still factors with roles in the complex process of melanomagenesis that are not yet fully understood [12]. Broadening the knowledge of the complex polygenic inheritance and molecular mechanisms underpinning the development of melanoma as well as identifying individuals at high risk of developing the disease are crucial starting points for improving prevention and early detection, which currently remains the best treatment option for the disease. High-risk individuals are also more likely to have multiple primary melanoma (MPM) diagnoses or several affected relatives in the same parental branch (familial melanoma, FM). It is estimated that about 8.2% of melanoma patients develop melanoma again during their lifetime and that about 5–10% of all melanoma cases have at least a first- or second-degree relative affected [13,14,15]. These high-risk patients are suspected to have a genetic background predisposing them to melanoma and should be offered genetic counseling and then genetic testing. In countries with a low–medium incidence of melanoma, like Italy, having two relatives with cutaneous melanoma and/or other related cancers (i.e., pancreatic cancer) or two primary melanoma diagnoses is a sufficient criterion for access to genetic melanoma predisposition tests [16].

Among the high-penetrance genes that affect melanoma risk, *CDKN2A* is the one that most frequently presents pathogenic variants in FM and MPM cases and, with its *CDK4* binding partner, was the first gene to be identified [17]. An Italian Melanoma Intergroup (IMI) familial melanoma study by Bruno et al. demonstrated that Italian families with at least two affected first-degree members carried a *CDKN2A* germline mutation with a frequency of 33% [18]. In addition, in a subsequent IMI study focused on multiple melanomas, *CDKN2A* germline mutations were found in 4.4% of patients with a single primary melanoma (SPM) and in 19% of MPM cases, regardless of family history. The prevalence of CDKN2A variants increased from 2.1% to 24.6% in SPM and from 10.8% to 44.4% in MPM without and with a positive melanoma family history, respectively. Focusing on the different trends across Italian regions [19], in the Veneto region, *CDKN2A* mutations occurred only in a small number (8,5%) of familial melanoma cases with at least two relatives affected by melanoma, suggesting the contribution of other genetic factors to melanoma susceptibility [20]. On the other hand, in agreement with previous studies [18,19,20,21], in Veneto, the presence of at least one MPM case in melanoma-prone families increased the frequency of pathogenic *CDKN2A* variants to 31.6% [20].

Over the last few decades, the advent of next-generation sequencing (NGS) technology has allowed for the identification of novel, rare high-risk variants in other susceptibility genes, such as *BAP1*, which is involved in cell-cycle control; *MITF*, which is involved in different stages of melanocyte development and differentiation [22]; *ATM*, which is involved in DNA damage response [23]; and *TERT*, *POT1*, *TERF2IP*, and *ACD*, which are implicated in the telomere maintenance pathway [24,25,26,27,28]. Nevertheless, less than 25% of genetic predisposition can be explained by high-penetrance genetic variants (approximately 19% by *CDKN2A* and 3% by other genes) [17,29,30]. Furthermore, in Italy, a pathogenic variant in high- or medium-penetrance genes was detected in only 9% of 273 Italian *CDKN2A*-negative melanoma cases [29].

Recent genome-wide association studies (GWASs) [31,32,33,34,35] have identified many common genetic variants (single nucleotide polymorphisms, SNPs) with a modifying effect on melanoma susceptibility. Although the effect sizes for any given disease-associated SNP are typically small and of limited predictive power, using the polygenic risk score (PRS) approach, the individual effect of multiple SNPs can be aggregated by summing the effect of the risk alleles as estimated by GWAS data. A recent genome-wide association meta-analysis of 36,760 cases of cutaneous melanoma identified 85 cutaneous melanoma susceptibility loci [35]. Most lead SNPs in these loci have been associated with nevogenesis (i.e., in *MTAP*, *PLA2G6*, *ASIP* genes), telomere maintenance (i.e., in *OBFC1*, *PARP1*, and *FTO* genes), and pigmentation (i.e., *MC1R*, *OCA2*, *TYR*, and *SLC45A2* genes); some of them are associated with more than one trait. These SNPs associated with cutaneous melanoma susceptibility and involved in different biological pathways [31,33,34,36] highlight the complexity of cutaneous melanoma etiology, also reinforcing the importance of identifying potential new pathways for cutaneous melanoma pathogenesis.

The utility of the PRS in clinical practice has already been demonstrated for other cancers, such as coronary artery disease (CAD), diabetes (types 1 and 2), obesity (and body mass index), prostate cancer, and Alzheimer’s disease [37]. The PRS was also incorporated into breast cancer risk prediction models such as BOADICEA [38], and commercial panels including SNPs are now available [39].

Regarding melanoma, in a study utilizing data from a large meta-analysis of melanoma GWASs of the Melanoma Meta-Analysis Consortium (MMAC), Gu et al. described a 204-SNP PRS model with an AUC of 64.4%. The PRS improved risk prediction by 1.4% (AUC = 69%) in an Italian cohort compared to a model based on established phenotypic risk factors alone [40]. The study of Wong et al. also combined a PRS with traditional clinical risk factors (such as skin type, sun exposure), showing a more comprehensive and accurate way to predict an individual’s melanoma risk [41]. Moreover, Potjer et al. investigated whether a polygenic risk score (PRS) was associated with melanoma risk in genetically unexplained cases with a family history, finding that a 46-SNP PRS was significantly associated with melanoma risk, with an AUC of 0.77 [42].

However, the inclusion of a PRS test in clinical practice is not yet considered appropriate by the American College of Medical Genetics and Genomics (ACMG) [43], so further evidence is needed. For this reason, we evaluated the effect of a 57-SNP PRS (PRS_57_) on melanoma risk in a cohort of 270 patients from the Veneto region in Italy with a suspected genetic melanoma predisposition but with a non-informative melanoma genetic test result for high/medium-penetrance genes.

## 2. Materials and Methods

### 2.1. Study Population

Among patients who were referred to oncogenetic counseling at the Familial Melanoma Clinic of the Veneto Institute of Oncology (IOV) in Padova between October 2019 and March 2023, 270 melanoma cases with a non-informative genetic test for high/medium-penetrance genes (i.e., *CDKN2A*, *CDK4*, *POT1*, *BAP1*, *ATM*, *MITF*, *ACD*, *TERT*) were selected (Table 1).

These melanoma patients had a suspected genetic predisposition to melanoma due to their familial or personal history of this disease (two affected first-degree relatives in the same familial branch or MPM cases) or for other related cancers (i.e., pancreatic adenocarcinoma, uveal melanoma, mesothelioma, renal cancer). At the last follow-up date, 93 patients were SPM cases and 177 were MPM cases. Information about phenotypic features, sunburns, and nevus counts were collected during counseling for almost all the cases according to a consensus questionnaire developed to standardize epidemiologic and clinical data collection for melanoma risk assessment [44].

A control series was used consisting of 296 healthy individuals without a personal or family history of melanoma enrolled at the Blood Collection Centre, Hospital Transfusion Centre of Padova. These controls had complete SNP data for PRS_57_ calculation, as they had already undergone genome-wide genotyping for a previous study [35].

Written informed consent was obtained from all participants of this study under local ethics committee-approved protocols.

### 2.2. Single Nucleotide Polymorphism (SNP) Analysis

The 270 cases were genotyped through NGS analysis, performed using a QIAseq Custom Targeted DNA Panel (Qiagen, Hilden, Germany) based on amplicon technology. The panel included high/medium-penetrance genes (i.e., *CDKN2A*, exon 2 of *CDK4*, *POT1*, *BAP1*, *ATM*, exon 9 of *MITF*, *ACD*, promoter region and exon 1 of *TERT*) and SNPs previously identified via GWAS (prior to 2020) [31,33,34,40]. A total of 1454 amplicons (of these, 1393 covering genes) were sequenced using a Mi-Seq instrument (Illumina, San Diego, CA, USA).

Data analysis was performed using the CLC Genomic Workbench software (Qiagen, Hilden, Germany) to generate genotype data for all SNPs per sample. In brief, the FastQ sequences were aligned to human reference genome GRCh37; variant calling, with a minimum read depth of 30×, was subsequently performed to produce a genomic VCF (gVCF) for each sample that was finally analyzed using the Identify QIAseq DNA Germline Variants pipeline (Qiagen, Hilden, Germany). After quality control (QC), we first removed ambiguous SNPs and those with a low pair-wise LD (maximum r2 of 0.45) and then obtained the genotype data of 57 SNPs (Supplementary Table S1) passing the quality parameters set according to the manufacturer’s instructions.

### 2.3. Polygenic Risk Score (PRS) Calculation

PRS_57_ was calculated for both healthy controls and melanoma cases using the following formula:

PRS_57_ = β1x1 + β2x2 + β3x3… + β57x57 (1)

βi is the estimated per-allele log OR for melanoma risk associated with the alternative allele at the ith SNP, taken from the most recent melanoma GWAS from the GenoMEL consortium [35], and xi is the number of alternative alleles carried by each individual, being 0 for those homozygous for the reference allele, 1 for heterozygotes, and 2 for those homozygous for the alternative allele. The PRS_57_ was then standardized to the mean and SD in the control series; before standardization, the mean PRS_57_ in population controls was 1.48 with an SD of 0.64.

### 2.4. Statistical Analysis

Quantitative variables were summarized as median or mean and quartiles or standard deviation (SD); categorical variables were reported as counts and percentages. The overall distribution of the PRS_57_ was compared among different groups using the Kruskall–Wallis test.

The PRS_57_, as a categorical variable, was first analyzed for association with melanoma risk using deciles of controls as cut-off points, and then for association with MPM risk using deciles of cases as cut-off points, considering the 5–6th and the 1st decile, respectively, as references. The relationship between the PRS_57_ and the probability of developing melanoma was evaluated by univariate logistic regression and adjusted for age at sampling and sex. The univariate and the adjusted models were specified as follows, respectively:

logit(p) = β0 + β1PRS_57_ (2)

logit(p) = β0 + β1PRS_57_ + β2age + β3sex (3)

where p represents the probability of the outcome occurring, β0 is the intercept, and β1, β2, and β3 are the coefficients for the predictors.

To quantify the effect size of the PRS_57_ independent variable, the crude and adjusted odds ratios (ORs) were reported with their 95% confidence intervals (CIs). In order to verify the discriminative ability of the PRS_57_, after adjusting for age at sampling and sex, the area under the curve (AUC) was estimated and compared between models with and without confounding factors using the DeLong’s test.

Similarly, the relationship between the melanoma risk factors, PRS_57_, and the probability of developing more than one melanoma was estimated using univariate logistic regression models. The univariate models were specified as follows:

logit(p) = β0 + β1X1(4)

where p represents the probability of the outcome occurring, X1 represents the independent variable, β0 is the intercept, and β1 the coefficient for the predictor. To quantify the effect size of each independent variable, the crude odds ratios (ORs) were reported with their 95% confidence intervals (CIs).

The independent role of each melanoma risk factor was verified in a multivariable logistic model considering all characteristics significantly associated with the outcome in the univariate analyses.

In order to verify the discriminative ability of PRS_57_, the area under the curve (AUC) was then estimated and compared between multivariable models with and without PRS_57_ using the DeLong’s test.

All tests were two-sided, and a *p*-value < 0.05 was considered statistically significant. No adjustment for multiple testing was performed. Statistical analyses were performed using RStudio (RStudio: Integrated Development for R. RStudio, Inc., Boston, MA, USA) and SAS version 9.4 (SAS Institute, Cary, NC, USA).

## 3. Results

### 3.1. PRS and Risk of Developing Melanoma

**Figure 1:**
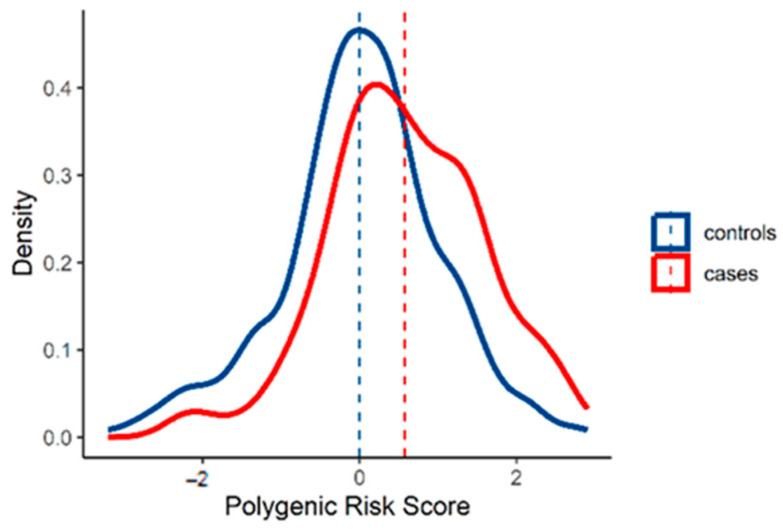
Smoothed kernel density estimates of the standardized PRS_57_ in all cases and controls. Dotted lines correspond to the means of the PRS_57_.

The genotypes of the 57 selected SNPs were used to calculate the PRS for each of the 270 melanoma cases with a suspected predisposition to melanoma but without pathogenic variants in high/medium-penetrance genes and 296 controls. Comparing overall melanoma cases vs. healthy controls, we observed a significant difference in the PRS_57_ distribution (Table 2, Figure 1). On average, melanoma cases had a significantly higher PRS_57_ than healthy controls, with a mean PRS_57_ of 0.577 (SD = 0.990).

**Figure 2:**
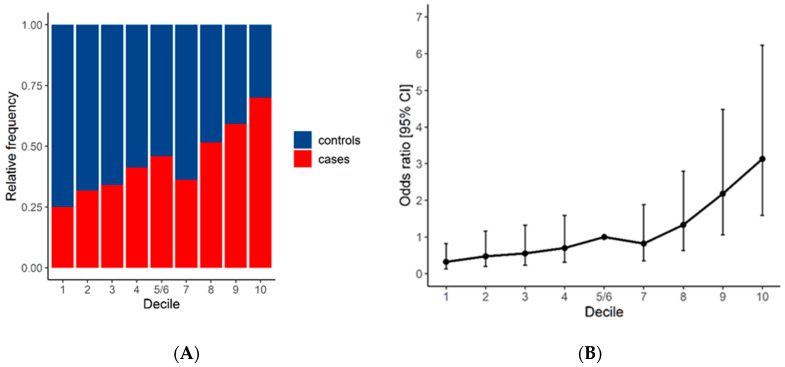
(**A**) Distribution of controls and cases in each decile; (**B**) ORs (points) with 95% CIs (vertical bars) by decile for melanoma cancer risk, using the 5–6th decile as the reference.

We then assigned melanoma cases to decile groups of the PRS_57_ distribution determined using the control series (Figure 2A). In a logistic regression model including sex and age at sampling as possible confounders, we observed that a higher melanoma risk was significantly associated with the highest PRS_57_ deciles. In particular, compared with those in the 5–6th decile, individuals with a PRS_57_ in the 9th and 10th deciles had an adjusted OR of 2.18 (95% CI 1.06 to 4.48) and 3.13 (95% CI 1.58 to 6.23) for developing melanoma, respectively. In contrast, the lower decile categories had a lower risk of melanoma, with an adjusted OR of 0.32 (95% CI 0.12 to 0.82, Table 3). We also observed a monotonic increase in risk with increasing PRS_57_ (Figure 2B).

**Figure 3:**
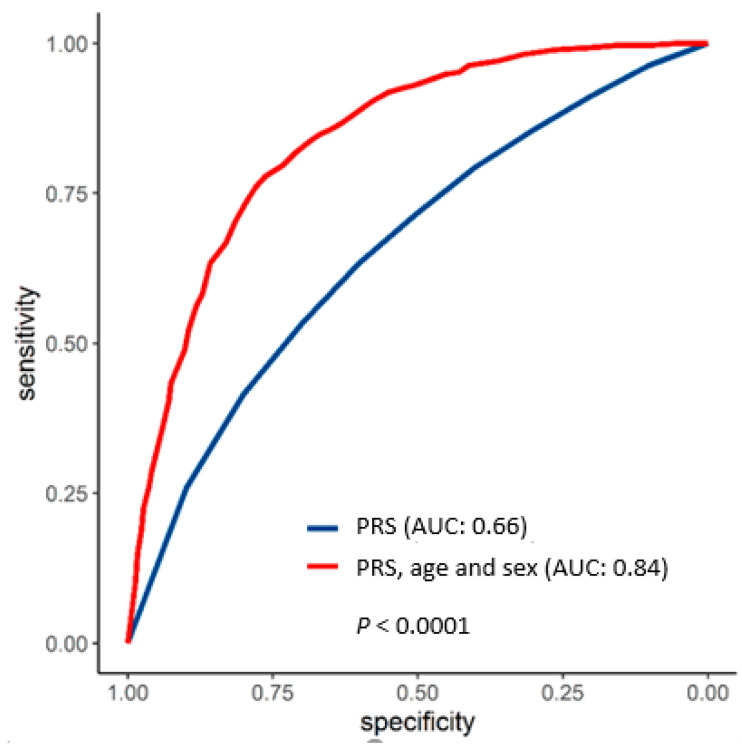
Area under the curve (AUC) for the prediction of developing melanoma based on PRS_57_ (categorized according to deciles) (blue) and a model based on PRS_57_, with age at sampling and sex included as possible confounders (red). *p*-value for the difference between the two AUCs (DeLong’s test).

The univariate PRS_57_ model had an AUC of 0.66, which significantly increased to 0.84 when age at sampling and sex were included (Figure 3).

### 3.2. PRS, Melanoma Risk Factors, and Risk of Developing Multiple Melanoma

Additional information on well-established melanoma risk factors such as eye/hair/skin color, sunburn frequency, and nevus count were available only for cases and not controls. Nevertheless, we observed a nominally significant association between PRS_57_ and almost all the features considered in the analysis (Table 4). Indeed, melanoma patients with light phenotypic colors, high sunburn frequency, and a young age at diagnosis had a significantly higher PRS_57_. In particular, individuals with light eyes were twice as likely to have a mean PRS_57_ higher than those with dark eyes, and individuals with red hair or skin phototype I–II had an almost three-fold higher PRS_57_ than those with dark hair or skin phototype III–IV, respectively. On the other hand, PRS_57_ was not significantly associated with the number of nevi, nor did PRS_57_ differ between patients with or without affected relatives. Interestingly, the mean PRS_57_ in the multiple melanoma group (0.689) was twice that in the single melanoma group (0.362) (Table 4).

**Figure 4:**
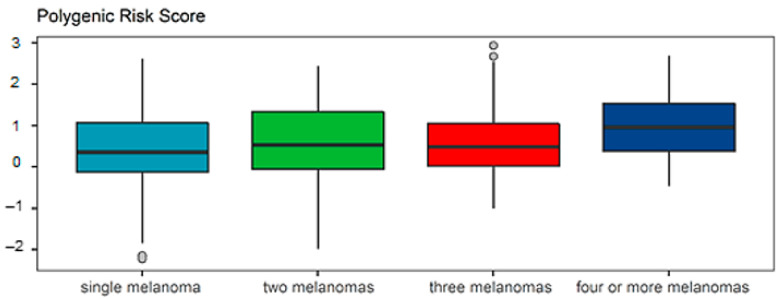
Box plot showing the different distributions between cases with one, two, three, or four or more melanomas. The bottom and the top of the box correspond to the first and third quartiles, and the bold-black line in the middle represents the median. The upper/lower whiskers extend to the largest/smallest value no further than 1.5× the inter-quartile range, or the distance between the first and third quartiles. Data beyond the end of the whiskers are outlying points.

In particular, a total of 93 patients with one melanoma had a median PRS_57_ of 0.322, which increased to 0.498 in 78 cases with two melanomas and 0.451 in 40 cases with three melanomas, up to 0.925 in 59 patients with four or more melanomas, with a *p*-value of 0.005 (Figure 4).

Then, we evaluated the impact that other melanoma risk factors considered so far could have on the risk of developing MPM. Our results were indicative of a higher risk of developing more than one melanoma during the lifetime in individuals with more than 50 nevi and in males. We also assigned melanoma cases to decile groups of the PRS_57_ distribution determined using the case series to explore if the risk of developing MPM increased with an increase in PRS_57_. We found that patients with a PRS_57_ in the 5th, 8th, 9th, and 10th deciles had a higher risk of MPM, especially those in the top decile, having an OR of 4.74 (Table 5).

**Figure 5:**
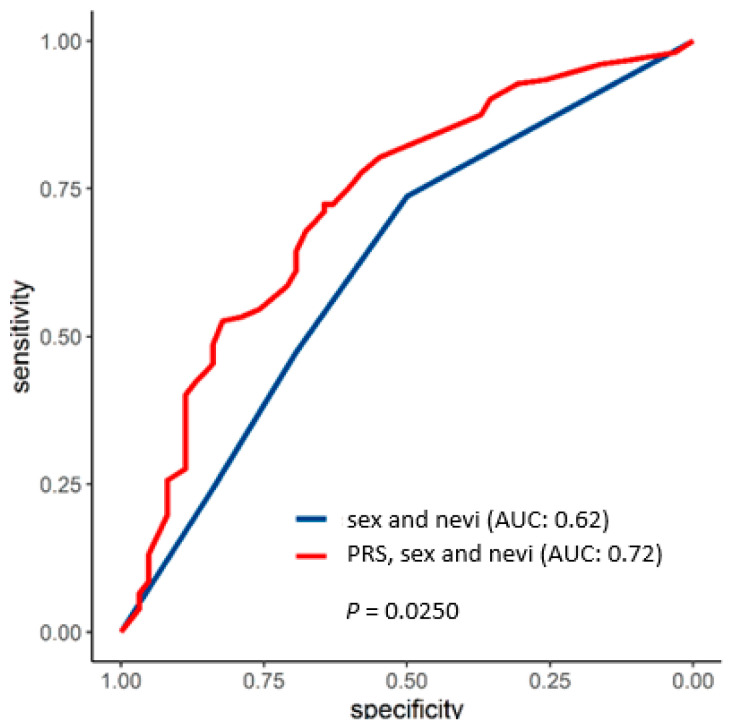
Area under the curve (AUC) for the prediction of developing multiple melanoma using either established risk factors for melanoma (sex and nevi (>50, <50)) (blue) or sex, nevi, and PRS_57_ (categorized according to deciles) (red).

It is noteworthy that the inclusion of the PRS_57_ in the risk prediction model, together with the risk factors that reached statistical significance from the univariate logistic regression analysis, significantly improved the discrimination of MPM-prone individuals, with the AUC increasing from 0.62 to 0.72 (*p*-value < 0.0250) (Figure 5).

## 4. Discussion

In this study, we defined a PRS based on 57 SNP genotypes that was found to be significantly associated with melanoma risk in a cohort of 270 melanoma patients with a suspected genetic predisposition but with a non-informative genetic test in high/medium-penetrance melanoma genes. Indeed, melanoma cases had a higher PRS_57_ than controls, and individuals in the top decile of the PRS_57_ distribution were more at a more that than three-fold higher risk of having melanoma than individuals in the middle decile (5–6th decile).

When studying only the cases, we first observed a significantly higher PRS_57_ in patients with an earlier age at melanoma diagnosis (51 vs. >51 years), suggesting a genetic background for melanoma susceptibility, and second, our results are indicative of a significant association of PRS_57_ with established risk factors for melanoma, such as multiple diagnoses of melanoma, pigmentary traits, and number of sunburns, but not with nevus count or family history. In particular, MPM cases had a significantly higher mean PRS_57_ than SPM cases, and the PRS_57_ increased proportionally with the increase in the number of melanomas until reaching a mean PRS_57_ value of 0.975 in individuals with more than four melanomas. On the other hand, the lack of a significant association of PRS_57_ with family history could be explained by considering that not only our familial cases but also the selected sporadic cases have a suspected genetic predisposition to melanoma. In fact, about 76% of the sporadic patients were MPM cases, and the remainder had a personal or familial (at least one first/second-degree relative) history of melanoma-related neoplasms (e.g., pancreatic carcinoma, mesothelioma). Thus, in our cohort of patients, the distinction between familial and sporadic melanoma is much less clear.

Then, we focused on assessing the impact of the factors considered so far on the risk of developing multiple melanoma. Among the cases, individuals with PRS_57_ values in the highest deciles (8th, 9th, 10th) had a higher risk of having MPM than SPM. In addition, when the PRS_57_ was considered a continuous variable, we observed that the per-SD OR increased to 1.41 for MPM. Consistent with our findings, Potjer et al. reported a per-SD OR increase to 2.86 for multiple primary melanomas, suggesting that individuals with a high PRS are not only more likely to develop melanoma but also to develop subsequent melanomas [42].

We also confirmed the nevus count as an important risk factor for the development of MPM. It is noteworthy that the PRS_57_, in addition to nevus count and sex, significantly improved the discrimination between SPM and MPM (AUC 0.62 vs. 0.72, *p* = 0.0250).

Our patients could be at high risk for disease through the combined effect of multiple common, low-risk genetic variants, reflected in a high PRS and high-risk phenotypes (such as pale pigmentation and propensity to sunburn). Otherwise, as already hypothesized [45], a second scenario could exist wherein the risk of melanoma is potentially less strongly modified by PRS and other risk factors because of the presence of high/medium-penetrance variants. This hypothesis agrees with previous findings that showed a lower PRS in cases of families carrying high-penetrance variants than in cases of families without [46] and in cases of families with high density compared to those with low density [42].

A major strength of our study is that it considered a group of patients strongly suspected of having a genetic background predisposing them to melanoma development despite an absence of pathogenic variants in high/medium-penetrance melanoma genes. Further studies in large cohorts of individuals carrying pathogenetic variants in melanoma genes could instead clarify whether and to what extent the PRS could modify the penetrance of such a variant.

Another advantage of this study is the inclusion of some traditional risk factors in our analysis to explore their possible association with the PRS. We confirmed an association between PRS and traditional risk factors, with higher PRS values for individuals with light eyes, red or blonde hair, skin phototype I–II, and frequent sunburns. Notably, individuals with a high nevus count, a risk factor associated with nearly double the likelihood of more than one melanoma, were more likely to have higher PRS values. These findings agree with a previous study that investigated the association between a 204-SNP PRS and traditional risk factors such as age, sex, skin phototype, number of nevi, and eye and hair color, genotyping for the first time individuals from a Mediterranean population. Importantly, it showed that this PRS strongly differentiated the melanoma risk in an Italian population in combination with the other melanoma risk factors, enabling the stratification of subjects into high- and low-risk groups [40].

However, it is still unclear to what degree genomic and traditional risk factors overlap in their prediction of melanoma risk, and further studies are needed. The main challenge remains how to determine which SNPs should be included in the PRS calculation and the weighting parameters (β values) to assign.

One of the limitations of our study is the small sample size, as this was a single-center investigation. This constraint complicates the broader interpretation of our findings and may limit their generalizability to larger populations. However, this pilot study has the advantage of a consistent and uniform data collection process, which enhances the reliability of the results obtained. The insights gained from this research will serve as a foundation for future studies with a wider cohort. Such subsequent research will not only validate our findings but also allow for a more comprehensive exploration of the associations identified in this study, thereby contributing to a deeper understanding of the underlying factors influencing melanoma. Another limitation of this study is that genetic analysis cannot represent the entire Italian population, considering the non-homogeneous prevalence of mutations in susceptibility genes, in particular *CDKN2A*, due to the geographical origin of the patients. Currently, this is still an intrinsic limitation of PRS validity, as the majority of studies in the PRS field are based on GWAS data from individuals with European ancestry, with little benefit for minority ancestry groups [47]. There are currently no guidelines for the inclusion of PRS in clinical practice, and the implementation of this approach must be carefully examined, especially for its ethical implications [43,48,49,50]. Avoiding misleading communication is crucial for appropriate genetic counseling and PRS testing, and the patient should be correctly informed on how the PRS results will be used in clinical management. Importantly, it should be clear that the PRS provides a relative and not an absolute risk of developing the considered disease. Therefore, an individual with a high PRS may never develop the disease, and individuals with low PRS might. Nevertheless, as genetic and phenotypic data increase in size, variety, and accuracy, the clinical utility and validity of the PRS will improve, especially through collaborations with other research groups within national and international consortia in a global effort.

## 5. Conclusions

In conclusion, our findings highlight the role of PRS as an additional useful tool in clinical practice to identify subjects at an elevated risk of developing not only melanoma but also multiple melanomas, especially in the context of a suspected melanoma predisposition but with a non-informative test for high/medium-penetrance genes.

It is noteworthy that some traditional risk factors were included in our analysis to assess their possible association with the PRS. This could lead to the definition of an individual’s genomic profile, which could further refine risk prediction models for this disease, considering that all the subjects with a high PRS should undergo more stringent protocols of surveillance for earlier detection and thus prompt treatment.

## Figures and Tables

**Table 1:** Characteristics of the study population. N/A = data missing because not collected.

		Cases	Controls
Number		270	296
Age at sampling	Mean (SD)	56.76 (13.42)	41.12 (12.08)
	Median (Q1, Q3)	56.00 (48.00, 66.00)	42.00 (36.00, 49.00)
Age at diagnosis	Mean (SD)	49.59 (14.90)	-
	Median (Q1, Q3)	51.00 (40.00, 59.00)	-
Sex	F	155 (57.4%)	82 (27.7%)
	M	115 (42.6%)	214 (72.3%)
Eye color	Dark	111 (47.0%)	-
	Light	125 (53.0%)	-
	N/A	34	-
Hair color	Brown/black	127 (53.8%)	-
	Blonde	88 (37.3%)	-
	Red	21 (8.9%)	-
	N/A	34	-
Fitzpatrick skin phototype	I–II	125 (53.0%)	-
	III–IV	111 (47.0%)	-
	N/A	34	-
Sunburn frequency	Rarely/never	103 (44.2%)	-
	Often	130 (55.8%)	-
	N/A	37	-
Nevi	<50	115 (53.7%)	-
	>50	99 (46.3%)	-
	N/A	56	-
Family history of melanoma	Yes	163 (60.4%)	-
	No	107 (39.6%)	-
No. of primary melanomas	=1	93 (34,4%)	-
	>1	177 (65,6%)	-

**Table 2:** Polygenic risk score (PRS) distribution by melanoma risk. *p*-value from Kruskall–Wallis test.

	Polygenic Risk Score (PRS)		
	Mean (SD)	Median (Q1, Q3)	*p*-Value
Cases (N = 270)	0.577 (0.990)	0.551 (−0.075, 1.301)	<0.001
Controls (N = 296)	0.000 (1.000)	0.041 (−0.505, 0.584)	

**Table 3:** The ORs (95% CIs) of each decile for melanoma risk were estimated from logistic regression using the 5–6th decile as reference, and the ORs * (95% CIs) were adjusted including age at sampling and sex.

		Cases (N = 270)	Controls (N = 296)	OR (95%CI)	OR * (95%CI)	*p*-Value
PRS_57_	1	10 (3.7%)	30 (10.1%)	0.39 (0.18, 0.89)	0.32 (0.12, 0.82)	0.017
	2	14 (5.2%)	30 (10.1%)	0.55 (0.26, 1.16)	0.47 (0.19, 1.16)	0.102
	3	15 (5.6%)	29 (9.8%)	0.61 (0.29, 1.27)	0.55 (0.23, 1.32)	0.181
	4	21 (7.8%)	30 (10.1%)	0.83 (0.42, 1.63)	0.70 (0.31, 1.58)	0.389
	5–6	49 (18.1%)	58 (19.6%)	ref.	ref.	
	7	17 (6.3%)	30 (10.1%)	0.67 (0.33, 1.36)	0.82 (0.35, 1.88)	0.636
	8	32 (11.9%)	30 (10.1%)	1.26 (0.67, 2.36)	1.33 (0.63, 2.79)	0.450
	9	42 (15.6%)	29 (9.8%)	1.71 (0.93, 3.15)	2.18 (1.06, 4.48)	0.033
	10	70 (25.9%)	30 (10.1%)	2.76 (1.56, 4.90)	3.13 (1.58, 6.23)	0.001

**Table 4:** Polygenic risk score (PRS) distribution by patient characteristics. *p*-value from Kruskall–Wallis test.

		Polygenic Risk Score (PRS)		
		Mean (SD)	Median (Q1, Q3)	*p*-Value
Age at diagnosis	≤51 (N = 141)	0.731 (1.048)	0.827 (0.002, 1.454)	0.002
	>51 (N = 129)	0.408 (0.895)	0.381 (−0.156, 0.924)	
Sex	F (N = 155)	0.484 (1.055)	0.450 (−0.168, 1.257)	0.100
	M (N = 115)	0.701 (0.884)	0.640 (0.099, 1.329)	
Eye color	Dark (N = 111)	0.349 (1.027)	0.317 (−0.258, 0.962)	<0.001
	Light (N = 125)	0.798 (0.900)	0.810 (0.165, 1.454)	
Hair color	Brown/black (N = 127)	0.376 (0.958)	0.462 (−0.158, 1.030)	0.001
	Blonde (N = 88)	0.741 (0.923)	0.561 (0.064, 1.513)	
	Red (N = 21)	1.216 (1.064)	1.397 (0.193, 1.916)	
Fitzpatrick skin phototype	I–II (N = 125)	0.850 (0.972)	0.886 (0.143, 1.517)	<0.001
	III–IV (N = 111)	0.303 (0.917)	0.318 (−0.211, 0.855)	
Sunburn frequency	Rarely/never (N = 103)	0.321 (0.947)	0.318 (−0.211, 1.017)	<0.001
	Often (N = 130)	0.798 (0.973)	0.726 (0.138, 1.468)	
Nevi	<50 (N = 115)	0.468 (1.006)	0.485 (−0.142, 1.153)	0.075
	>50 (N = 99)	0.723 (0.990)	0.719 (−0.017, 1.450)	
Family history of melanoma	Yes (N = 163)	0.546 (0.971)	0.556 (−0.049, 1.228)	0.683
	No (N = 107)	0.623 (1.020)	0.485 (−0.089, 1.450)	
No. of melanomas	=1 (N = 93)	0.362 (1.042)	0.322 (−0.156, 1.031)	
	>1 (N = 177)	0.689 (0.944)	0.597 (0.002, 1.390)	0.025

**Table 5:** The ORs (95% CIs) for MPM were estimated from univariate logistic regression models.

		MPM (N = 177)	SPM (N = 93)	OR (95%CI)	*p*-Value
Age at diagnosis	≤51	97 (54.8%)	44 (47.3%)	ref.	0.242
	>51	80 (45.2%)	49 (52.7%)	0.74 (0.45, 1.23)	
Sex	F	93 (52.5%)	62 (66.7%)	ref.	0.027
	M	84 (47.5%)	31 (33.3%)	1.81 (1.07, 3.05)	
Eye color	Dark	81 (49.7%)	30 (41.1%)	ref.	0.222
	Light	82 (50.3%)	43 (58.9%)	0.71 (0.4, 1.23)	
Hair color	Brown/black	83 (50.9%)	44 (60.3%)	0.83 (0.47, 1.49)	0.544
	Blonde	61 (37.4%)	27 (37.0%)	ref.	
	Red	19 (11.7%)	2 (2.7%)	4.2 (0.91, 19.33)	0.065
Fitzpatrick skin phototype	I–II	90 (55.2%)	35 (47.9%)	ref.	0.302
	III–IV	73 (44.8%)	38 (52.1%)	0.75 (0.43, 1.3)	
Sunburns frequency	Rarely/never	67 (41.6%)	36 (50.0%)	0.71 (0.41, 1.25)	0.234
	Often	94 (58.4%)	36 (50.0%)	ref.	
Nevi	<50	75 (49.3%)	40 (64.5%)	ref.	0.045
	>50	77 (50.7%)	22 (35.5%)	1.87 (1.01, 3.43)	
PRS_57_	Cont.			1.41 (1.08, 1.83)	0.011
PRS_57_	1	13 (7.3%)	14 (15.1%)	ref.	
	2	18 (10.2%)	9 (9.7%)	2.15 (0.72, 6.47)	0.172
	3	18 (10.2%)	9 (9.7%)	2.15 (0.72, 6.47)	0.172
	4	14 (7.9%)	13 (14.0%)	1.16 (0.40, 3.37)	0.786
	5	21 (11.9%)	6 (6.5%)	3.77 (1.16, 12.27)	0.028
	6	17 (9.6%)	10 (10.8%)	1.83 (0.62, 5.42)	0.275
	7	13 (7.3%)	14 (15.1%)	1.00 (0.34, 2.91)	1.000
	8	21 (11.9%)	6 (6.5%)	3.77 (1.16, 12.27)	0.028
	9	20 (11.3%)	7 (7.5%)	3.08 (0.98, 9.67)	0.054
	10	22 (12.4%)	5 (5.4%)	4.74 (1.39, 16.21)	0.013

## Data Availability

Data that support the findings of this study are openly available from Zenodo at http://doi.org/10.5281/zenodo.13767214 (accessed on 16 September 2024).

## Supplementary Materials

The following supporting information can be downloaded at: https://www.mdpi.com/article/10.3390/biology13110954/s1, Table S1: List of 57 single nucleotide polymorphisms (SNPs).

## References

[1] Damsky W.E., Bosenberg M. (2017). Melanocytic Nevi and Melanoma: Unraveling a Complex Relationship. Oncogene.

[2] Slominski R.M., Sarna T., Płonka P.M., Raman C., Brożyna A.A., Slominski A.T. (2022). Melanoma, Melanin, and Melanogenesis: The Yin and Yang Relationship. Front. Oncol..

[3] Whiteman D.C., Watt P., Purdie D.M., Hughes M.C., Hayward N.K., Green L.C. (2003). Melanocytic Nevi, Solar Keratoses, and Divergent Pathways to Cutaneous Melanoma. J. Natl. Cancer Inst..

[4] Ferrara G., Argenziano G. (2021). The WHO 2018 Classification of Cutaneous Melanocytic Neoplasms: Suggestions From Routine Practice. Front. Oncol..

[5] Sinclair C., Foley P. (2009). Skin Cancer Prevention in Australia. Br. J. Dermatol..

[6] Sung H., Ferlay J., Siegel R.L., Laversanne M., Soerjomataram I., Jemal A., Bray F. (2021). Global Cancer Statistics 2020: GLOBOCAN Estimates of Incidence and Mortality Worldwide for 36 Cancers in 185 Countries. CA A Cancer J. Clin..

[7] Briatico G., Mancuso P., Argenziano G., Longo C., Mangone L., Moscarella E., Brancaccio G., Pampena R. (2022). Trends in Cutaneous Melanoma Mortality in Italy from 1982 to 2016. Int. J. Dermatol..

[8] Gandini S., Sera F., Cattaruzza M.S., Pasquini P., Abeni D., Boyle P., Melchi C.F. (2005). Meta-Analysis of Risk Factors for Cutaneous Melanoma: I. Common and Atypical Naevi. Eur. J. Cancer.

[9] Gandini S., Sera F., Cattaruzza M.S., Pasquini P., Picconi O., Boyle P., Melchi C.F. (2005). Meta-Analysis of Risk Factors for Cutaneous Melanoma: II. Sun Exposure. Eur. J. Cancer.

[10] Gandini S., Sera F., Cattaruzza M.S., Pasquini P., Zanetti R., Masini C., Boyle P., Melchi C.F. (2005). Meta-Analysis of Risk Factors for Cutaneous Melanoma: III. Family History, Actinic Damage and Phenotypic Factors. Eur. J. Cancer.

[11] Rastrelli M., Tropea S., Rossi C.R., Alaibac M. (2014). Melanoma: Epidemiology, Risk Factors, Pathogenesis, Diagnosis and Classification. Vivo.

[12] Slominski R.M., Kim T.-K., Janjetovic Z., Brożyna A.A., Podgorska E., Dixon K.M., Mason R.S., Tuckey R.C., Sharma R., Crossman D.K. (2024). Malignant Melanoma: An Overview, New Perspectives, and Vitamin D Signaling. Cancers.

[13] Soura E., Eliades P.J., Shannon K., Stratigos A.J., Tsao H. (2016). Hereditary Melanoma: Update on Syndromes and Management: Genetics of Familial Atypical Multiple Mole Melanoma Syndrome. J. Am. Acad. Dermatol..

[14] Toussi A., Mans N., Welborn J., Kiuru M. (2020). Germline Mutations Predisposing to Melanoma. J. Cutan. Pathol..

[15] Cornish D., Holterhues C., van de Poll-Franse L.V., Coebergh J.W., Nijsten T. (2009). A Systematic Review of Health-Related Quality of Life in Cutaneous Melanoma. Ann. Oncol..

[16] American Society of Clinical Oncology (2003). American Society of Clinical Oncology Policy Statement Update: Genetic Testing for Cancer Susceptibility. J. Clin. Oncol..

[17] Potrony M., Badenas C., Aguilera P., Puig-Butille J.A., Carrera C., Malvehy J., Puig S. (2015). Update in Genetic Susceptibility in Melanoma. Ann. Transl. Med..

[18] Bruno W., Ghiorzo P., Battistuzzi L., Ascierto P.A., Barile M., Gargiulo S., Gensini F., Gliori S., Guida M., Lombardo M. (2009). Clinical Genetic Testing for Familial Melanoma in Italy: A Cooperative Study. J. Am. Acad. Dermatol..

[19] Landi M., Goldstein A., Tsang S., Munroe D., Modi W., Ter-Minassian M., Steighner R., Dean M., Metheny N., Staats B. (2004). Genetic Susceptibility in Familial Melanoma from Northeastern Italy. J. Med. Genet..

[20] Menin C., Vecchiato A., Scaini M.C., Elefanti L., Funari G., De Salvo G.L., Quaggio M., Tognazzo S., Agata S., Santa S.D. (2011). Contribution of Susceptibility Gene Variants to Melanoma Risk in Families from the Veneto Region of Italy. Pigment. Cell Melanoma Res..

[21] Bruno W., Pastorino L., Ghiorzo P., Andreotti V., Martinuzzi C., Menin C., Elefanti L., Stagni C., Vecchiato A., Rodolfo M. (2016). Multiple Primary Melanomas (MPMs) and Criteria for Genetic Assessment: MultiMEL, a Multicenter Study of the Italian Melanoma Intergroup. J. Am. Acad. Dermatol..

[22] Levy C., Khaled M., Fisher D.E. (2006). MITF: Master Regulator of Melanocyte Development and Melanoma Oncogene. Trends Mol. Med..

[23] Dalmasso B., Pastorino L., Nathan V., Shah N.N., Palmer J.M., Howlie M., Johansson P.A., Freedman N.D., Carter B.D., Beane-Freeman L. (2021). Germline ATM Variants Predispose to Melanoma: A Joint Analysis across the GenoMEL and MelaNostrum Consortia. Genet. Med..

[24] Vinagre J., Almeida A., Pópulo H., Batista R., Lyra J., Pinto V., Coelho R., Celestino R., Prazeres H., Lima L. (2013). Frequency of TERT Promoter Mutations in Human Cancers. Nat. Commun..

[25] Flynn R.L., Zou L. (2010). Oligonucleotide/Oligosaccharide-Binding (OB) Fold Proteins: A Growing Family of Genome Guardians. Crit. Rev. Biochem. Mol. Biol..

[26] Robles-Espinoza C.D., Harland M., Ramsay A.J., Aoude L.G., Quesada V., Ding Z., Pooley K.A., Pritchard A.L., Tiffen J.C., Petljak M. (2014). POT1 Loss-of-Function Variants Predispose to Familial Melanoma. Nat. Genet..

[27] Shi J., Yang X.R., Ballew B., Rotunno M., Calista D., Fargnoli M.C., Ghiorzo P., Bressac-de Paillerets B., Nagore E., Avril M.F. (2014). Rare Missense Variants in POT1 Predispose to Familial Cutaneous Malignant Melanoma. Nat. Genet..

[28] Aoude L.G., Pritchard A.L., Robles-Espinoza C.D., Wadt K., Harland M., Choi J., Gartside M., Quesada V., Johansson P., Palmer J.M. (2015). Nonsense Mutations in the Shelterin Complex Genes ACD and TERF2IP in Familial Melanoma. J. Natl. Cancer Inst..

[29] Pastorino L., Andreotti V., Dalmasso B., Vanni I., Ciccarese G., Mandalà M., Spadola G., Pizzichetta M.A., Ponti G., Tibiletti M.G. (2020). Insights into Genetic Susceptibility to Melanoma by Gene Panel Testing: Potential Pathogenic Variants in ACD, ATM, BAP1, and POT1. Cancers.

[30] Leachman S.A., Lucero O.M., Sampson J.E., Cassidy P., Bruno W., Queirolo P., Ghiorzo P. (2017). Identification, Genetic Testing, and Management of Hereditary Melanoma. Cancer Metastasis Rev..

[31] Bishop D.T., Demenais F., Iles M.M., Harland M., Taylor J.C., Corda E., Randerson-Moor J., Aitken J.F., Avril M.-F., Azizi E. (2009). Genome-Wide Association Study Identifies Three Loci Associated with Melanoma Risk. Nat. Genet..

[32] Amos C.I., Wang L.-E., Lee J.E., Gershenwald J.E., Chen W.V., Fang S., Kosoy R., Zhang M., Qureshi A.A., Vattathil S. (2011). Genome-Wide Association Study Identifies Novel Loci Predisposing to Cutaneous Melanoma. Hum. Mol. Genet..

[33] Barrett J.H., Iles M.M., Harland M., Taylor J.C., Aitken J.F., Andresen P.A., Akslen L.A., Armstrong B.K., Avril M.-F., Azizi E. (2011). Genome-Wide Association Study Identifies Three New Melanoma Susceptibility Loci. Nat. Genet..

[34] Barrett J.H., Taylor J.C., Bright C., Harland M., Dunning A.M., Akslen L.A., Andresen P.A., Avril M.-F., Azizi E., Bianchi Scarrà G. (2015). Fine Mapping of Genetic Susceptibility Loci for Melanoma Reveals a Mixture of Single Variant and Multiple Variant Regions. Int. J. Cancer.

[35] Landi M.T., Bishop D.T., MacGregor S., Machiela M.J., Stratigos A.J., Ghiorzo P., Brossard M., Calista D., Choi J., Fargnoli M.C. (2020). Genome-Wide Association Meta-Analyses Combining Multiple Risk Phenotypes Provide Insights into the Genetic Architecture of Cutaneous Melanoma Susceptibility. Nat. Genet..

[36] Law M.H., Bishop D.T., Lee J.E., Brossard M., Martin N.G., Moses E.K., Song F., Barrett J.H., Kumar R., Easton D.F. (2015). Genome-Wide Meta-Analysis Identifies Five New Susceptibility Loci for Cutaneous Malignant Melanoma. Nat. Genet..

[37] Lambert S.A., Abraham G., Inouye M. (2019). Towards Clinical Utility of Polygenic Risk Scores. Hum. Mol. Genet..

[38] Yiangou K., Kyriacou K., Kakouri E., Marcou Y., Panayiotidis M.I., Loizidou M.A., Hadjisavvas A., Michailidou K. (2021). Combination of a 15-SNP Polygenic Risk Score and Classical Risk Factors for the Prediction of Breast Cancer Risk in Cypriot Women. Cancers.

[39] Lewis C.M., Vassos E. (2020). Polygenic Risk Scores: From Research Tools to Clinical Instruments. Genome Med..

[40] Gu F., Chen T.H., Pfeiffer R.M., Fargnoli M.C., Calista D., Ghiorzo P., Peris K., Puig S., Menin C., De Nicolo A. (2018). Combining Common Genetic Variants and Non-Genetic Risk Factors to Predict Risk of Cutaneous Melanoma. Hum. Mol. Genet..

[41] Wong C.K., Dite G.S., Spaeth E., Murphy N.M., Allman R. (2023). Melanoma Risk Prediction Based on a Polygenic Risk Score and Clinical Risk Factors. Melanoma Res..

[42] Potjer T.P., van der Grinten T.W.J., Lakeman I.M.M., Bollen S.H., Rodríguez-Girondo M., Iles M.M., Barrett J.H., Kiemeney L.A., Gruis N.A., van Asperen C.J. (2021). Association between a 46-SNP Polygenic Risk Score and Melanoma Risk in Dutch Patients with Familial Melanoma. J. Med. Genet..

[43] Abu-El-Haija A., Reddi H.V., Wand H., Rose N.C., Mori M., Qian E., Murray M.F. (2023). The Clinical Application of Polygenic Risk Scores: A Points to Consider Statement of the American College of Medical Genetics and Genomics (ACMG). Genet. Med..

[44] Stratigos A., Fargnoli M., De Nicolo A., Peris K., Puig S., Soura E., Menin C., Calista D., Ghiorzo P., Mandala M. (2018). MelaNostrum: A Consensus Questionnaire of Standardized Epidemiologic and Clinical Variables for Melanoma Risk Assessment by the Melanostrum Consortium. J. Eur. Acad. Dermatol. Venereol..

[45] Pellegrini S., Elefanti L., Dall’Olmo L., Menin C. (2021). The Interplay between Nevi and Melanoma Predisposition Unravels Nevi-Related and Nevi-Resistant Familial Melanoma. Genes.

[46] Schlafly A., Pfeiffer R.M., Nagore E., Puig S., Calista D., Ghiorzo P., Menin C., Fargnoli M.C., Peris K., Song L. (2019). Contribution of Common Genetic Variants to Familial Aggregation of Disease and Implications for Sequencing Studies. PLoS Genet..

[47] Martin A.R., Kanai M., Kamatani Y., Okada Y., Neale B.M., Daly M.J. (2019). Clinical Use of Current Polygenic Risk Scores May Exacerbate Health Disparities. Nat. Genet..

[48] Yanes T., McInerney-Leo A.M., Law M.H., Cummings S. (2020). The Emerging Field of Polygenic Risk Scores and Perspective for Use in Clinical Care. Hum. Mol. Genet..

[49] Adeyemo A., Balaconis M.K., Darnes D.R., Fatumo S., Granados Moreno P., Hodonsky C.J., Inouye M., Kanai M., Kato K., Knoppers B.M. (2021). Responsible Use of Polygenic Risk Scores in the Clinic: Potential Benefits, Risks and Gaps. Nat. Med..

[50] Slunecka J.L., van der Zee M.D., Beck J.J., Johnson B.N., Finnicum C.T., Pool R., Hottenga J.-J., de Geus E.J.C., Ehli E.A. (2021). Implementation and Implications for Polygenic Risk Scores in Healthcare. Hum. Genom..

