# Supplementary material for: Polygenic Risk Score Improves Melanoma Risk Assessment in a Patient Cohort from the Veneto Region of Italy
Source: Biology (Basel). 2024 Nov 20;13(11):954. doi: 10.3390/biology13110954 (PMC11592222; doi:10.3390/biology13110954)
Supplement: Supplementary file 1 [file biology-13-00954-s001.zip › biology-3274205-supplementary.pdf]

Supplementary Table S1. List of 57 Single Nucleotide Polymorphisms (SNPs), selected for the Polygenic Risk Score (PRS) calculation, with the most recent Beta value estimates.

	**SNP ID**	**Chromosome**	**Region/Gene**	**Reference allele**	**Alternative allele**	**Beta**
1	rs3768013	1	ARNT	G	A	-0,0765
2	rs1858550	1	PARP1	C	A	-0,0836
3	rs6750047	2	RMDN2/CYP1B1	A	G	-0,053
4	rs4670813	2	CYP1B1	G	A	-0,0647
5	rs7582362	2	FLACC1	A	G	-0,062
6	rs7632095	3	LOC102724419	A	G	0,03036
7	rs12696304	3	TERC/MYNN	C	G	-0,0618
8	rs2282679	4	GC	T	G	-0,0091
9	rs75724758	4	INPP4B	T	C	0,04594
10	rs380286	5	CLPTM1L	G	A	0,12738
11	rs16891982	5	SLC45A2	C	G	0,6298
12	rs250417	5	SLC45A2	G	C	0,62811
13	rs72767211	5	ANKRD55	C	T	0,00632
14	rs11738706	5	TENM2	C	T	0,06774
15	rs12203592	6	IRF4	C	T	0,13984
16	rs9405705	6	IRF4	C	G	0,04909
17	rs6914598	6	CDKAL1	T	C	0,0836
18	rs9257445	6	KRT18P1/ZNF311	G	C	-0,002
19	rs72909003	6	LOC105377871	G	A	0,10959
20	rs1636744	7	AGR3	C	T	0,05774
21	rs62444470	7	intergenic	T	C	0,13365
22	rs661356	9	DOCK8	G	A	-0,0412
23	rs72706189	9	TYRP1	T	C	0,02809
24	rs11532907	9	MTAP	A	G	-0,1195

25	rs77283072	9	CDKN2B-AS1	G	A	0,09069
26	rs10739221	9	TMEM38B	T	C	-0,0959
27	rs7041168	9	RNA5SP294	G	T	-0,07
28	rs2995264	10	OBFC1/STN1	G	A	-0,1398
29	rs2290419	11	CCND1	A	G	-0,06
30	rs498136	11	CCND1	A	C	-0,0811
31	rs12422135	11	CCND1	A	G	-0,0688
32	rs1126809	11	TYR	G	A	0,19784
33	rs10830253	11	TYR	T	G	0,17483
34	rs73008229	11	ATM	G	A	-0,1164
35	rs1640875	12	GPRC5A	A	T	0,08023
36	rs184628474	14	TTC7B	G	A	-0,0344
37	rs4778138	15	OCA2	A	G	-0,1405
38	rs7164220	15	OCA2	C	A	0,13628
39	rs145720174	15	HERC2	G	A	-0,2505
40	rs74456670	15	LYSMD2	A	G	0,03874
41	rs12596638	16	FTO	G	A	0,08447
42	rs11648879	16	FANCA	A	G	0,11227
43	rs75570604	16	FANCA	G	C	0,44504
44	rs35158985	16	CDH1	A	G	0,07268
45	rs1805005	16	MC1R	G	T	-0,0055
46	rs1805006	16	MC1R	C	A	0,40407
47	rs2228479	16	MC1R	G	A	0,10358
48	rs11547464	16	MC1R	G	A	0,32712
49	rs1805007	16	MC1R	C	T	0,47209
50	rs1110400	16	MC1R	T	C	0,16111

51	rs1805008	16	MC1R	C	T	0,32296
52	rs885479	16	MC1R	G	A	0,06454
53	rs1805009	16	MC1R	G	C	0,37411
54	rs6060627	20	BCL2L1	C	T	-0,0301
55	rs6088372	20	RALY	C	T	0,23332
56	rs6059655	20	RALY	A	G	-0,3416
57	rs132985	22	PLA2G6	C	T	-0,0809
